# High-resolution mapping and breeding application of a novel brown planthopper resistance gene derived from wild rice (*Oryza. rufipogon* Griff)

**DOI:** 10.1186/s12284-019-0289-7

**Published:** 2019-06-04

**Authors:** Zhihua Li, Yanxia Xue, Hailian Zhou, Yang Li, Babar Usman, Xiaozhen Jiao, Xinyi Wang, Fang Liu, Baoxiang Qin, Rongbai Li, Yongfu Qiu

**Affiliations:** 1 State Key Laboratory for Conservation and Utilization of Subtropical Agro-bioresources, Agricultural College, Guangxi University, Nanning, 530005 China; 2 School of Electrical and Control Engineering, North University of China, Taiyuan, 030051 China

**Keywords:** *Oryza rufipogon* Griff, Brown planthopper (*Nilaparvata lugens* Stål), Resistance gene, Gene mapping, Marker-assisted selection, Near isogenic line, Pyramided line

## Abstract

**Background:**

The brown planthopper (*Nilaparvata lugens* Stål; BPH), one of the most destructive pests of rice, has proven to be a substantial threat, conferring enormous production losses in Asia and becoming a difficult challenge to manipulate and control under field conditions. The continuous use of insecticides promotes the resurgence of BPH, which results in resistant varieties adapting through the upgrading of new BPH biotypes. To overcome resistance acquired by BPH against resistance varieties, different forms of novel resistant gene fusions act as functional domains for breeding to enhance insect resistance.

**Results:**

The current study reports on the novel BPH resistance gene *Bph36* derived from two introgression lines (RBPH16 and RBPH17) developed from wild rice GX2183 which was previously reported to be resistant to BPH. Using two F_2_ crossing populations (Kangwenqizhan × RBPH16 and Huanghuazhan × RBPH17) in a bulked segregant analysis (BSA) for identification of resistant genes and QTL analysis, two QTLs for BPH resistance were generated on the long and short arms of chromosome 4, which was further confirmed by developing BC_1_F_2:3_ populations by backcrossing via marker assisted selection (MAS) approach. One BPH resistance locus on the short arm of chromosome 4 was mapped to a 38-kb interval flanked by InDel markers S13 and X48, and then was named *Bph36*, whereas another locus on the long arm of chromosome 4 was also detected in an interval flanked by RM16766 and RM17033, which was the same as that of *Bph27*. An evaluation analysis based on four parameters (BPH host selection, honeydew weight, BPH survival rate and BPH population growth rate) shows that *Bph36* conferred high levels antibiosis and antixenosis to BPH. Moreover, *Bph36* pyramided with *Bph3*, *Bph27*, and *Bph29* through MAS into elite cultivars 9311 and MH511 (harbored *Xa23*), creating different background breeding lines that also exhibited strong resistance to BPH in the seedling or tillering stage.

**Conclusion:**

*Bph36* can be utilized in BPH resistance breeding programs to develop high resistant rice lines and the high-resolution fine mapping will facilitate further map-based cloning and marker-assisted gene pyramiding of resistant gene. MAS exploited to pyramid with *Bph3*, *Bph27*, *Bph29*, and *Xa23* was confirmed the effectiveness for BPH resistance breeding in rice and provided insights into the molecular mechanism of defense to control this devastating insect.

**Electronic supplementary material:**

The online version of this article (10.1186/s12284-019-0289-7) contains supplementary material, which is available to authorized users.

## Background

The brown planthopper (*Nilaparvata lugens* Stål; BPH) is the most destructive insect pest of rice found throughout tropical Asia where rice is widely planted (Cha et al. 2008). The BPH causes direct damage to rice by sucking the plant’s sap and indirect damage by transmitting viral diseases such as grassy and ragged stunt viruses, resulting in severe yield reduction or complete crop losses referred as a result of the “hopper burn” phenomenon (Rivera et al. 1966; Ling et al. 1978; Sōgawa 1982; Normile 2008). According to the Statistics of China Agriculture Yearbook, over 25 million hectares of rice were infested with rice planthoppers in each year of 2005–2007. To manage the pest, the extensive use of chemical pesticides has generally worked well over the past decades, but the long term application of pesticides resulted in increased BPH resistance to chemical insecticides and presents the risk of destroying the environment (Heinrichs et al. 1982; Tanaka et al. 2000). However, ensuring the genetic resistance of host plants is the most effective and environmentally-friendly approach for the BPH management. Therefore, it is very necessary and imperative to detect more novel resistant genes and to then elucidate the resistance mechanism.

Thirty- five major BPH-resistance genes have been identified from cultivated rice and wild *Oryza* species (Wang et al. 2018). Wild rice species and their introgression lines have been progressively used as resistance harbors, and almost half of BPH resistance genes are derived from them (Ali and Chowdhury, 2014; Hu et al., 2016a, b). For example, resistance genes *Bph10* and *Bph18*(*t*) were detected from introgression lines of *O. australiensis* with the EE genome (Ishii et al. 1994; Jena et al. 2006). Resistance genes *bph11*(*t*), *bph12*(*t*), *Bph13*(*t*), *Bph14*, *Bph15*, and *QBph4.1* were identified from introgression lines of *O. officinalis* with the CC genome (Hirabayashi et al., 1998, 1999; Renganayaki et al. 2002; Du et al. 2009; Lv et al. 2014; Hu et al. 2015). Resistance gene *Bph12* was derived from the introgression line B14 of *O. latifolia* with the CCDD genome (Qiu et al. 2012). Resistance genes *Bph20*(*t*) and *Bph21*(*t*) were identified from *O. minuta* with the CCDD genome and were mapped to chromosomes 4 and 12, respectively (Rahman et al. 2009). Finally, *Bph27* and *Bph29* were identified from *O. rufipogon* with the AA genome (Huang et al. 2013; Wang et al. 2015). Considerable efforts have been made to clone major BPH-resistance genes, and a few successes have been achieved over the past ten years. For instance, *Bph14*, *Bph9*, and *Bph6* were successfully map-based cloned and to encode one type of NB-LRR protein (Du et al. 2009; Zhao et al. 2016; Guo et al. 2018). Resistance gene *Bph3*, a cluster of three repeated genes, encodes plasma membrane–localized lectin receptor kinases, confers broad-spectrum insect resistance and serves as a reference for the molecular breeding of insect-resistant cultivars (Liu et al., 2015). *Bph29* is a recessive gene derived from *O. rufipogon* and has been found to contain a B3 DNA-binding domain (Wang et al. 2015).

The interaction of plants and insects is complex and can be described in relation to physical, chemical, cellular, or molecular aspects. To limit damage from insects to plants, one may employ antixenosis, antibiosis, or insect tolerance through physiological functions (Kennedy et al. 1987; Alam and Cohen, 1998). Several BPH resistance genes have been demonstrated to confer one or more types of resistance mechanisms. For instance, Panda and Heinrichs (1983) determined that rice varieties carrying the *Bph1* gene exhibit antibiosis and tolerance and observed moderate resistance to the BPH. Moreover, resistance genes *Bph6* and *Bph14* have been characterized to have antibiotic and antixenotic effects on insects (Qiu et al. 2010; Du et al. 2009). Together, different BPH resistance genes may possess different resistance mechanisms. Thus, it is still necessary to demonstrate resistance mechanisms and to identify resistance effects observed when one novel BPH resistance gene is detected, which should facilitate resistant rice breeding.

The identified BPH resistance genes provide a wealth of resistance resources for rice breeding and improvement. However, it must be noted that the BPH insects can adapt to rice varieties with single resistance gene and then develop new biotypes for practical rice cultivation. For example, two BPH resistance varieties, IR26 and IR36, harboring BPH resistance genes *Bph1* and *bph2*, respectively, were widely cultivated in the 1970s and 1980s, but they have become susceptible in the last few years due to changes in BPH biotypes and infestation patterns (Claridge and Hollander, 1980; Pathak et al. 1969). Previous studies have indicated that rice lines pyramiding two or more BPH resistance genes via marker-assisted selection (MAS) can efficiently improve resistance levels (Qiu et al. 2012; Hu et al., 2016a, b; Jena et al. 2017). Thus, there is still an urgent need to pyramid multiple BPH resistance genes and to develop stronger and more durable varieties that are resistant to BPH.

GX2183, a wild species of *O. rufipogon* with the AA genome, shows broad spectrum resistance to BPH biotypes, including biotypes 1 and 2, Bangladesh (collected from Bangladesh), Cuu Long (collected from Vietnam), and Pantnagar (collected from India) (Li et al. 2006). Major resistance gene *Bph27* has been finely mapped to an 86.3-kb region harbored by markers RM16846 and RM16853 (Huang et al. 2013). Meanwhile, this study reveals the presence of more than one major BPH resistance gene in the rice species of GX2183. In the present study, introgression lines of GX2183 were used to develop the mapping population, near isogenic lines (NILs), and pyramiding lines (PYLs) to map and apply the BPH resistance gene. We identified and finely mapped a new BPH resistance gene *Bph36* flanked by InDel markers S13 and X48 on the short arm of rice chromosome 4 and incorporated this gene into the elite rice cultivars via MAS.

## Materials and methods

### Mapping population, NIL, and PYL development

RBPH16 and RBPH17, two introgression lines derived from *O. rufipogon* accession GX2183, are highly resistant to BPH and were used as the donor. Rice varieties Kangwenqingzhan (abbreviated as KW) and Huanghuazhan (abbreviated as HHZ) are widely cultivated in southern China and are highly susceptible to BPH. RBPH16 and RBPH17 were respectively crossed with KW and HHZ to develop F_2:3_ mapping populations. Based on the QTL analysis, the positive F_1_ (KW/RBPH16) individuals were continuously backcrossed with KW to developed BC_1_F_1_, BC_2_F_1_, BC_3_F_1_, and BC_4_F_1_ populations, and each backcrossed individual was verified with tightly linked markers. From the developed populations, single-gene individuals carrying the novel resistance gene were self-crossed twice to develop a BC_1_F_2_ population, and then they were used to verify the resistance gene and for fine mapping. Positive BC_4_F_1_ individuals were then self-crossed twice to obtain BC_4_F_2_ lines homozygous to the resistance genes and taken as NILs.

RBPH54, an introgression line derived from *O. rufipogon*, carries BPH-resistance gene *Bph29* (Wang et al. 2015). Rice variety PTB33 has been indicated to be highly resistant to BPH and includes resistance gene *Bph3* in the short arm of chromosome 4 (Liu et al., 2015). Both were used to develop PYLs with BPH resistance genes. MH511, carrying the *Xa23* resistance gene, is a bacterial blight resistant line. Rice line 9311 is a merit restoring line that is highly susceptible to BPH. To develop PYLs with different BPH resistance genes, RBPH16 was first crossed with RBPH54 and PTB33, and then the positive individuals were backcrossed with RBPH16 twice. Next, BC_2_F_1_ individuals derived from RBPH16/RBPH54 or RBPH16/PTB33 were crossed and self-crossed twice. Then, the BC_2_F_2_ lines were evaluated based on BPH resistance levels, and highly BPH- resistant lines were crossed with MH511 or 9311. Susceptible lines MH511 and9311 were taken as recurrent parents to develop BC_3_F_1_, and each backcrossed individual was verified with the tightly linked markers (Additional file 1: Figure S1).

Wild rice accession GX2183 was collected from Guangxi province of China and was stored at Guangxi University. Rice lines RBPH16, RBPH17, RBPH54, and MH511 were developed in our laboratory. Rice varieties 9311, KW, and HHZ were collected and stored in our laboratory as germplasm.

### BPH insects and evaluation of BPH resistance

The BPH insects were collected from rice fields in Nanning, Guangxi province of China, and were maintained on Taichung Native 1 (TN1, a susceptible *indica* variety) under natural conditions in a greenhouse at Guangxi University. A bulk seedling test was conducted to evaluate BPH resistance as described by Qiu et al. (2010). The pre-germinated seeds were sown in rows spaced 3.5 cm apart with 20 seeds in each line in a plastic box (58 × 38 × 7 cm) with a seed bed of 3-cm thick fine puddled soil. Susceptible control TN1, resistant control PTB33 and parents of the mapping population were sown in thecenter. Seedlings of the third-leaf stage (13 or 14 days old) were infested with second- to third-instar hopper nymphs at a density of eight insects per seedling. Temperature and humidity were respectively maintained at 28 ± 2 °C and 75 ± 5% for evaluation. Phenotypic values for the individual plants were recorded on a scale on 0–9 when all plants of susceptible control TN1 were killed. This was done following the Standard Evaluation System (SES) for rice (IRRI, 1996) based on the following scale: 0 = no visible damage; 1 = partial yellowing in first leaf; 3 = first and second leaves have become partially yellow; 5 = pronounced yellowing or minor stunting; 7 = wilting while, the plant is still alive; and 9 = the plant has completely wilted or died. Three replicates of each line were carried out, and all resistance tests were repeated twice.

### Marker analysis, QTL detection and gene mapping

Total genomic DNA of the plant leaves was extracted by the CTAB method (Murray and Thompson, 1980). PCR was performed as described by Qiu et al. (2010), and the PCR products were separated on 7% denaturing polyacrylamide gels and visualized by silver staining. Simple sequence repeat (SSR) primer sequences were obtained from the Gramene database (www.gramene.org/archive),and InDel makers were developed through a comparison of genome sequences of *japonica* cv. Nipponbare and *indica* cv. 9311.

A bulked segregant analysis (BSA) was performed to identify markers linked to BPH resistance (Michelmore et al. 1991). According to the results of our BPH resistance evaluation, ten extremely resistant and ten extremely susceptible individuals were used to construct two contrasting DNA pools. SSR and InDel markers were screened for polymorphism between the two pools. All polymorphic markers within the study region were used to genotype the F_2_ individuals. Genetic linkage maps of SSR and InDel markers with BPH resistance loci were constructed using JoinMap 3.0. Genetic linkage maps and phenotypes of the primary mapping population were used for QTL analysis via inclusive composite interval mapping (ICIM) with MapQTL 5.0. QTLs were identified when LOD scores exceeded 3.0.

### BPH host selection test

To perform the BPH host selection test, each rice line with a single gene (NIL-*Bph36*), two genes (NIL-*Bph36* + *Bph27*), and KW were sown into one row with 15 healthy seedlings (4 weeks old) in a plastic bucket containing rice field soil. The second- to third-instar BPH nymphs were released into an average of eight heads per plant. The number of BPH insects attached to each line was counted every day after infestation. After each observation, the base of each rice plant was gently tapped to allow BPH insects to be evenly distributed throughout the water and to select the host plant once again. Three replications were conducted each time and the test was repeated twice.

### BPH honeydew excretion measurement

The parafilm sachets method was used to measure BPH honeydew excretion (Pathak et al. 1982). One fifth-instar BPH insect was enclosed in a rectangular (3.5 cm × 3 cm) parafilm bag with a pipette, and then was fastened to the test plants (4 weeks old). Each plant had one or two bags and eight bags for each line of NIL-*Bph36*, NIL-*Bph36* + *Bph27*, or KW wereused. After 2 days of insect infestation, the bags were removed and the total weight of the bag enclosing the honeydew was weighed as W1. Then, the honeydew was wiped with absorbent cotton and the bag was weighed again (W2). Therefore, the weight of honeydew excretion was calculated as W1 – W2. The test was repeated three times.

### BPH survival and growth measurement

The BPH survival rate was determined according to the method described by Qiu et al. (2010). The 5-week-old seedlings were only composed of main stems and were covered with a plastic cup with a hole in the base. Then, each plant was infested with ten third-instar nymphs. The surviving insects were counted 1 d, 2 d, 3 d, 4 d, and 5 d after BPH release. Six plants were arranged for each line of NIL-*Bph36*, NIL-*Bph36* + *Bph27*, and KW, and the experiment was repeated three times.

To measure the population growth rate (PGR) of the BPH, the rice seedlings were treated as they were for the BPH survival test. The 5-week-old seedlings were infested with 15 pre-weighed second- to third-instar nymphs and then covered with a plastic cup with a hole sealed with absorbent cotton. The weight of the surviving BPH was measured after 4 days of insect infestation. The PGRs of BPH were calculated following the method described by Qiu et al. (2010). Six plants were used for each line of NIL-*Bph36*, NIL-*Bph36* + *Bph27*, and KW, and the test was repeated three times.

### Bacterial blight resistance test

To perform the bacterial blight resistance test, *Xanthomonas oryzae* pv*. oryzae* GX1070 was collected from rice plants from Guangxi province of China and was cultured on nutrient agar (NA) media at 28 °C for 4 days. The bacteria were then collected and dissolved with ddH_2_O to an optical density concentration of OD_600_ = 1.0. The 8-week-old plants were then inoculated with bacteria using the leaf-clipping method, and then each leaf was measured and scored after 3 weeks of inoculation (Kauffman et al. 1973). Six plants were used for each line, and the test was repeated twice.

### MAS for the resistance genes

According to the above study, molecular markers RHD9, RH007, and RHC10 were co-segregated with *Bph3* (Liu et al., 2015), and BYL8, BID2, BID3, and BYL7 were tightly linked to *Bph29* (Wang et al. 2015). *Bph27* was found in the region flanked by SSR markers RM16846 and RM16888, which were used for MAS (Huang et al. 2013). These markers were verified for RBPH16, RBPH54, PTB33, 9311, and MH511 to select suitable markers for MAS.

### Agronomic trait measurement

To detect agronomic traits of the developed BPH-resistance lines, each line was composed of five rows and each row included ten plants at a planting density of 20 × 30 cm (20-cm spacing between plants and 30-cm spacing between rows distance). Together with the recurrent parents, three repeats of each line were randomly distributed across the rice field. Then, 20 plants from each repeat were subjected to agronomic trait measurements, (i.e., plant height, effective panicle number, the number of grains per panicle, 1000-grain weight, and seed setting percentage). Field management followed normal agronomic practices. The test was repeated over two seasons.

### Data analysis

The Chi-square test for goodness-of-fit was performed with GraphPad Prism 7; and the resistance data were analyzed using one-way ANOVA and comparing the LSD test at a 5% or 1% significance level. The survival rates of insects (%) were arcsine transformed prior to analysis.

## Results

### Genetic analysis of BPH resistance

**Fig. 1:**
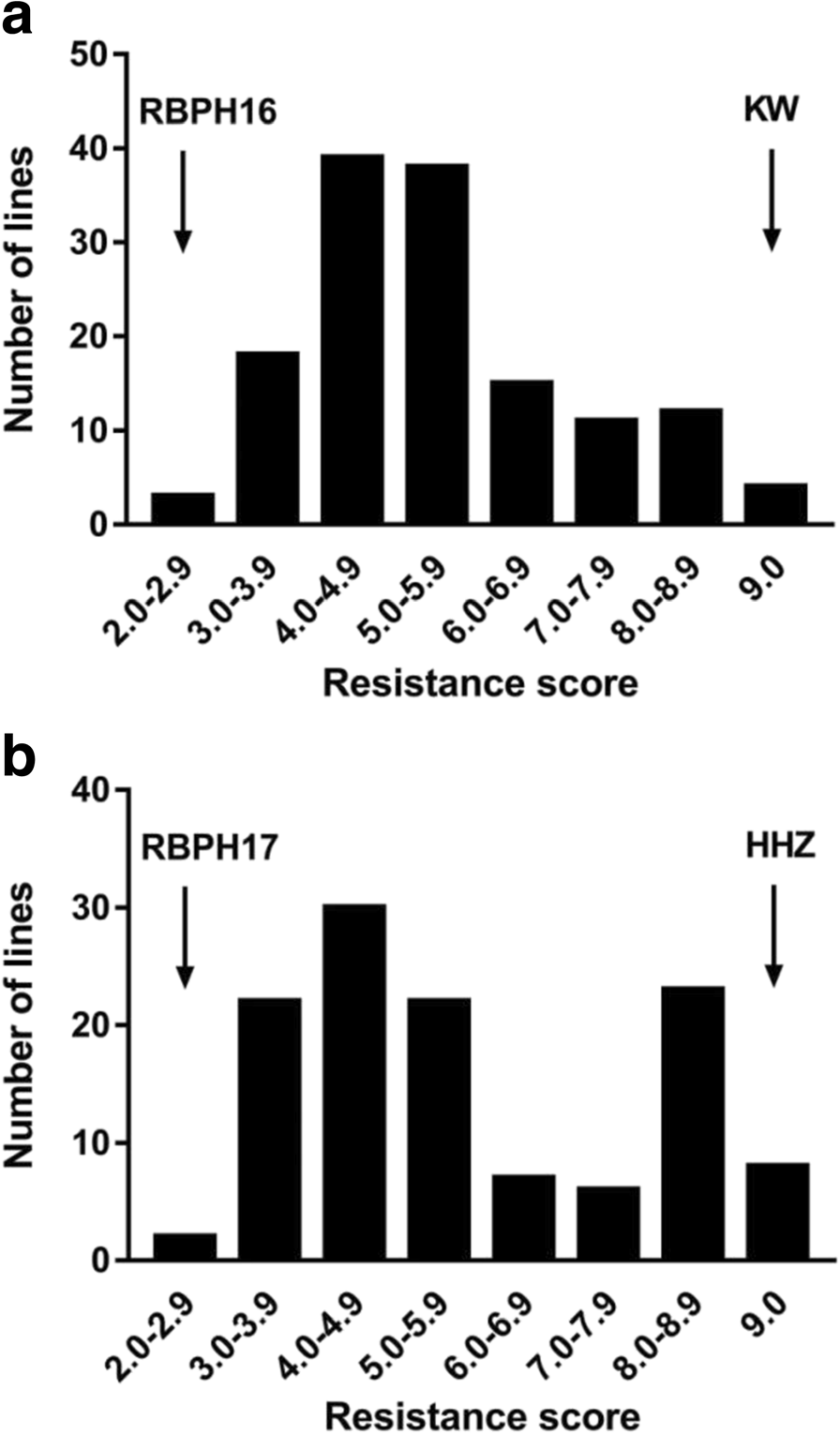
Frequency distribution of the BPH-resistance score in F_2_ population. **a** Population derived from cross of KW × RBPH16; **b** Population derived from cross of HHZ × RBPH17. Rice (*Oryza sativa*) seedlings were infested with eight BPHs per plant for 9–10 days. RBPH16 and RBPH17 were the resistance parents; KW (Kangwenqingzhan) and HHZ (Huanghuazhan) were the susceptible parents

A previous study showed that wild rice accession GX2183 (*O. rufipogon*) contains other BPH resistance genes except for *Bph27* (Huang et al. 2013). As it is relatively difficult to develop mapping populations directly crossing with GX2183, we obtained highly BPH-resistant introgression lines and then constructed the associated mapping population. As a result, introgression lines RBPH16 and RBPH17 were derived from GX2183 and were found to be highly resistant to BPH. Furthermore, *indica* rice cultivars KW and HHZ were found to be highly susceptible to BPH and were applied to develop mapping populations (Fig. 1). Then, F_2_ lines developed from two different mapping populations were surveyed based on resistance levels via SES. Consequently, the resistance scores of 140 individuals of the F_2_ population of the crossing KW/RBPH16 varied continuously from 2.0 to 9.0 with some lines showing super-progeny phenomena (Fig. 1a). According to the IRRI scoring criteria for seedling tests and previous studies conducted by Qiu et al. (2010), we considered resistance scores of 0–7.0 to denote resistance and of 7.1–9.0 to denote susceptibility. Therefore, the BPH resistant assay showed that the segregation of resistance to susceptibility in 140 F_2_ lines disagreed with 3:1 (113:27; χ2 C = 7.02 > χ2 0.05 = 3.84) (Fig. 1a). The same trend of phenotypic variance was observed for the other F_2_ population derived from HHZ/RBPH17. The segregation of resistance to susceptibility for 120 F_2_ lines also disagreed with 3:1 (75:45; χ2 C = 9.34 > χ2 0.05 = 3.84) (Fig. 1b). These results indicated that more than one major gene conferred BPH resistance in the introgression lines.

### Primary mapping of resistance genes

A BSA was performed to identify markers linked to the BPH resistance loci. According to the resistance score for F_2_ individuals of KW/RBPH16 population, a total of 968 molecular markers distributed across 12 rice chromosomes were used to screen polymorphism between extremely resistant and susceptible DNA bulks. As a result, six markers were found to be polymorphic, of which markers RM471, RM17033, and RM16874 and markers RM16502, RM518, and RM335 were identified from the contiguous region of the long and short arms of chromosome 4, respectively. The same analysis was conducted on the F_2:3_ mapping population of HHZ/RBPH17. A total of 952 markers were surveyed with the two DNA bulks. Markers RM17033, RM16888, RM16874, RM16858, and RM16766 from the long arm exhibited polymorphism, and markers RM335, RM518, RM16465, and RM16502 from the short arm exhibited polymorphism. We found two major resistance loci on chromosome 4. We then used the polymorphic markers to assay the F_2_ individuals and acquired a local linkage map developed by JoinMap3.0 (Table 1). Based on genotypes and phenotypes of detected F_2_ individuals within the two mapping populations, we analyzed the resistance loci using MapQTL5.0. As a result, two major loci with large LOD scores and with phenotypic variation were detected on chromosome 4. For mapping population KW/RBPH16, the locus on the long arm of chromosome 4 was detected with an interval flanked by RM16888 and RM16786 with a maximum LOD score of 14.4, contributing 38.2% of the phenotypic variation. The locus on the short arm of chromosome 4 was detected with an interval flanked by RM16465 and RM6659 with a maximum LOD score of 20.3, contributing 49.2% of the phenotypic variation (Table 1). For mapping population HHZ/RBPH17, the locus on the long arm of chromosome 4 was detected with an interval flanked by RM17033 and RM16786 with a maximum LOD score of 11.3, contributing 36.1% of the phenotypic variation. The locus on the short arm of chromosome 4 was detected with an interval flanked by RM16465 and RM16502 with a maximum LOD score of 31.1, contributing 86.8% of the phenotypic variation (Table 1). Taken together, these mapping results suggest that the same chromosome regions harbored BPH resistance loci according to an analysis of different F_2_ populations. According to a study conducted by Huang et al. (2013), the locus on the long arm of chromosome 4 is generally identical to resistant gene *Bph27*, while the locus on the short arm differs from the reported genes according to an analysis of genome information on the associate markers. Therefore, the locus harbored by RM16465 and RM16502 (or RM6659) was taken as a novel gene and was tentatively designated as *Bph36*.

**Table 1:** QTL scanning results using 140 lines (KW/RBPH16) and 120 lines (HHZ/RBPH17) resistance scores and marker genotypes of F_2_ population by MapQTL 5

Mapping population	Locus	Position (cM)	LOD	PEV (%)	*A*
KW/RBPH16-*Bph36*	RM335	0	0.6	1	− 0.2
	RM16465	9.1	0.9	0.7	−1.2
	*Bph36*	9.2	20.3	49.2	−1.8
	RM6659	10.2	20.0	48.4	−1.8
	RM16502	15.4	1.35	2.3	−0.2
	RM5412	18.7	0.4	0.7	−0.3
KW/RBPH16-*Bph27*	RM16766	0	1.7	3.4	0.6
	RM471	16.9	0.1	0.1	0
	RM16852	17.1	0	0	0.3
	RM16853	17.5	0.9	1.9	0.8
	*Bph27*	17.7	14.4	38.2	−1.5
	RM16858	18.7	13.5	35.8	−1.4
	RM16888	19.4	0.26	0.5	0.6
HHZ/RBPH17-*Bph36*	RM335	0	2.5	3.6	0.2
	RM518	5	2.1	3.3	0.1
	RM16465	13.9	4.5	54.3	−1.9
	*Bph36*	17.6	31.1	86.8	−2.2
	RM16502	20.6	19.7	9.1	−0.8
HHZ/RBPH17-*Bph27*	RM16766	0	0.8	2.2	−0.6
	RM16852	17	9.2	1.7	0.5
	RM16853	19.2	11.1	36.1	−1.6
	*Bph27*	20.2	11.8	36.6	−1.5
	RM16858	23.3	0.6	30.7	−1.5
	RM16874	24.7	0.1	0.2	0.3
	RM16888	25.5	0.2	0.4	0.4

PEV (%) Percentage of total phenotypic variance explained by the locus, *A* additive effect on the resistance allele

### Verification and fine mapping of *Bph36*

**Fig. 2:**
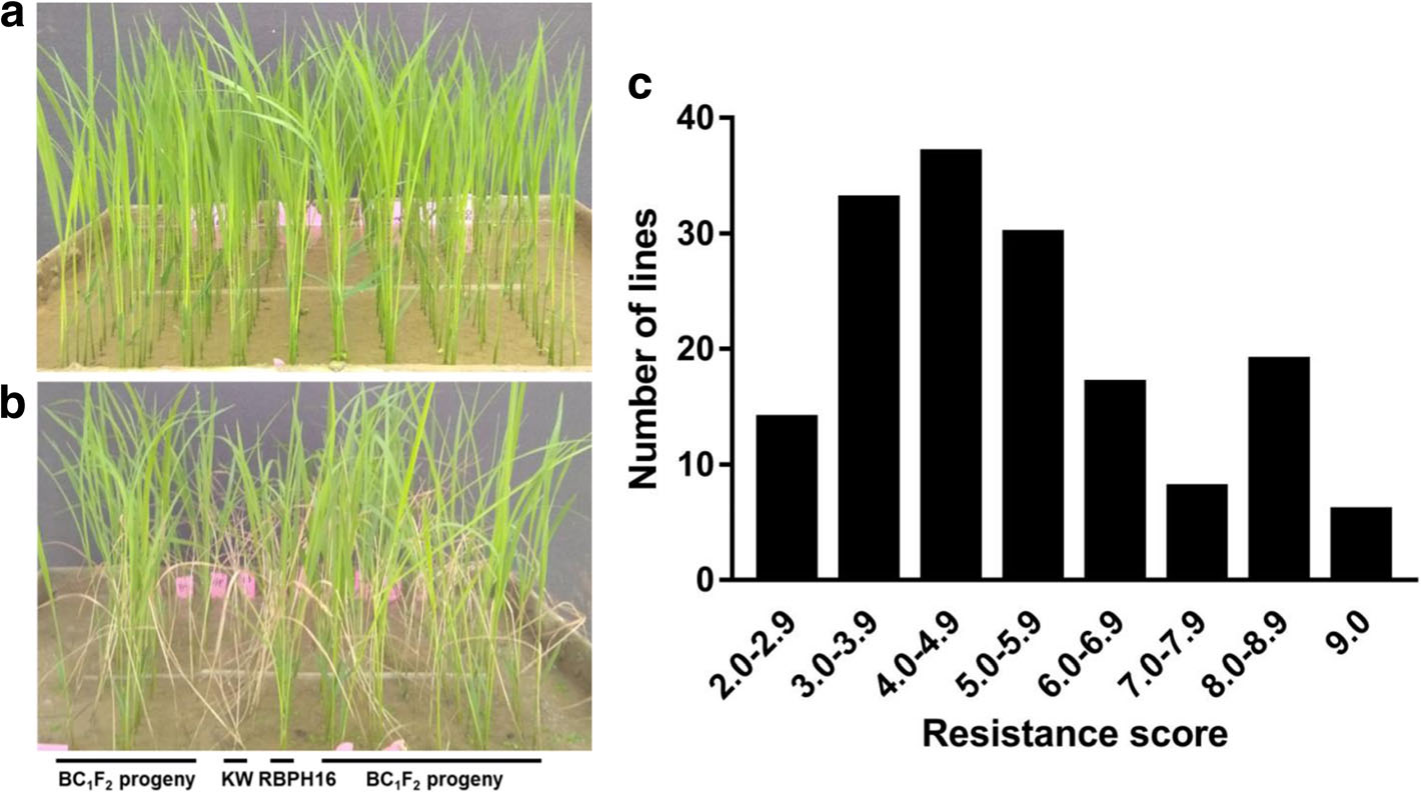
Overview of the bulk seedling test and frequency distribution of the BPH-resistance score in BC_1_F_2:3_ population carrying *Bph36*. **a** Before insect infestation of BC_1_F_2_ lines. **b** After insect infestation of BC_1_F_2_ lines. **c** Frequency distribution of the BPH-resistance score in BC_1_F_2_ population carrying *Bph36*. Rice seedlings were treated with eight BPHs per plant for 9–10 days. RBPH16 and KW were the resistance and susceptible parents, respectively

To confirm the genetic segregation of the chromosome region with the target gene, we only used BC_1_F_1_ individuals carrying *Bph36* to self-cross. Then the developed BC_1_F_2_ population derived from KW/RBPH16 was surveyed with a BPH seedling bulk test (Fig. 2a, b). Consequently, the BPH-resistance scores of 161 BC_1_F_2_ lines showed a continuous distribution of 2.0 to 9.0, and the segregation of resistant (0–7.0) and susceptible lines (7.1–9.0) presented an acceptable fit to a 3:1 ratio (123:38; χ2 C = 0.168 < χ2 0.05 = 3.84) (Fig. 2c). This result denotes the presence of a single gene determining BPH resistance in the selected mapping population.

**Fig. 3:**
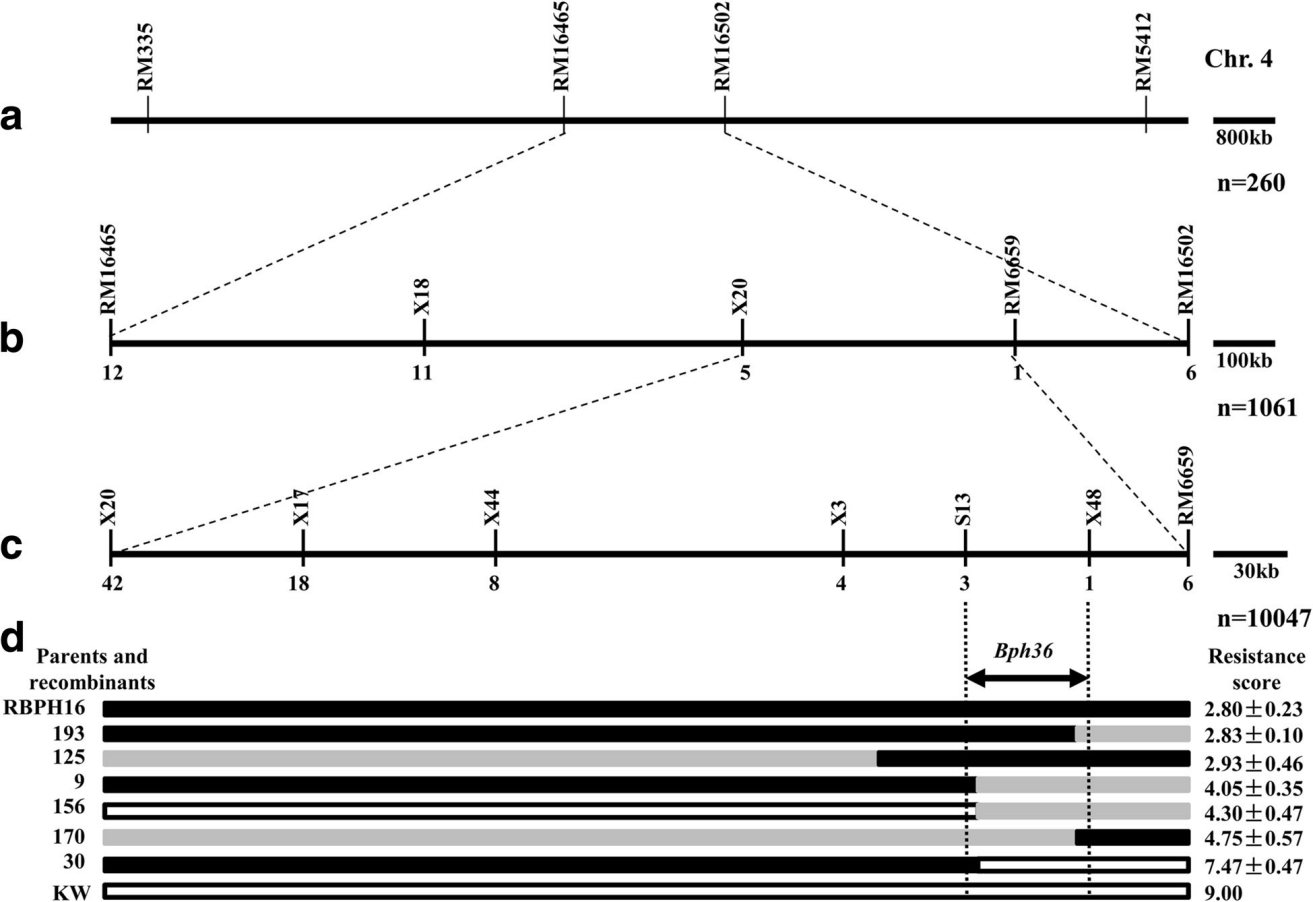
High-resolution mapping of *Bph36*. **a** Primary mapping of *Bph36* on the short arm of chromosome 4; **b** Fine mapping of *Bph36*; **c** High-resolution mapping of *Bph36*. The number below the lines denotes the number of recombinants between the lateral markers; **d** Graphical genotypes and resistance phenotypes of the recombinants. The black, white, and gray bars denote the marker genotypes of RBPH16 homozygotes, KW homozygotes, and heterozygotes, respectively

For the high-resolution mapping of the resistance gene, we conducted two rounds of fine mapping. We first developed polymorphic SSR and InDel markers X18, X20, RM6659, RM16465, and RM16502 between RM5412 and RM335 (Fig. 3a,b; Table 2). Then, a total of 1061 BC_1_F_3_ individuals from monogenic lines carrying *Bph36* were subjected to analysis and 18 recombinant lines were identified from RM16465 and RM6659. According to our analysis of genotypes and resistance scores of the recombinants, the resistance gene was identified in the region between X20 and RM6659 (Fig. 3b). Second, markers X20 and RM6659 were used to screen the genotypes of 10,047 BC_2_F_2_ seedlings derived from monogenic lines carrying *Bph36*. As a result, 68 recombinant individuals were identified from the selected markers (X20 and RM6659). At the same time, polymorphic InDel markers X3, X17, X44, X48, and S13 were developed in the region of interest (Table 2). Based on our analysis of genotypes and resistance scores of the recombinants, recombinants 170 and 193 could delimit the left margin of the gene with X48 and recombinants 9, 30, and 156 could delimit the right margin with S13. Eventually, we determined the candidate genomic region of *Bph36* for a 38-kb region flanked by markers S13 and X48 based on the genome of Nipponbare (Fig. 3c, d).

**Table 2:** Polymorphic InDel molecular markers used for fine mapping

ID	Forward primer (5′-3′)	Reverse primer (5′-3′)
X3	ATCCGTCCTTATCGTGTGCC	CCCTGTAGCCGATGGAGTTC
X17	GAGCGCAGGGAGTGGATTAG	GTCCAACATGATAGCGACCG
X18	CAGTCGGCCCAGTCATTGAT	GCATAAGTGAACAGAAGTGACCG
X20	TGCATACATCGATGAACAACCTC	ATTTGGCTTTTTCGTAGGGGT
X44	ACAACACAAACCTCAAGGAG	TCCCTTATTGCATTTACAGT
X48	CAAGGGAAACCAAACAGACAA	AAGACGACGGTTCTGGATTG
S13	AGCCCAAATGGAACAAACAA	GCTCACGGTTAAGCAATGGT

In consideration of the target region, four candidate genes were contained in the corresponding genomic region of Nipponbare. They are hypothetical protein (*LOC_Os04g11800*), acyltransferase family protein (*LOC_Os04g11810*), white-brown complex homolog protein (*LOC_Os04g11820*), and TCP family transcription factor (*LOC_Os04g11830*). Acyltransferase is a large family of proteins that play an important role in the biosynthesis of some plant secondary metabolites for defensing against insect (Chedgy et al. 2015). White-brown complex (WBC) family protein, is a part of ABC superfamily protein, has been reported to participate in a multitude of physiological processes that allow the plant to adapt to changing environments and cope with biotic and abiotic stresses (Kretzschmar et al. 2011). TCP family transcription factor participate in plant developmental processes, such as development of leaf and flower (Koyama et al. 2017; Yin et al. 2018).

### Characterization of the BPH resistance of NILs

**Fig. 4:**
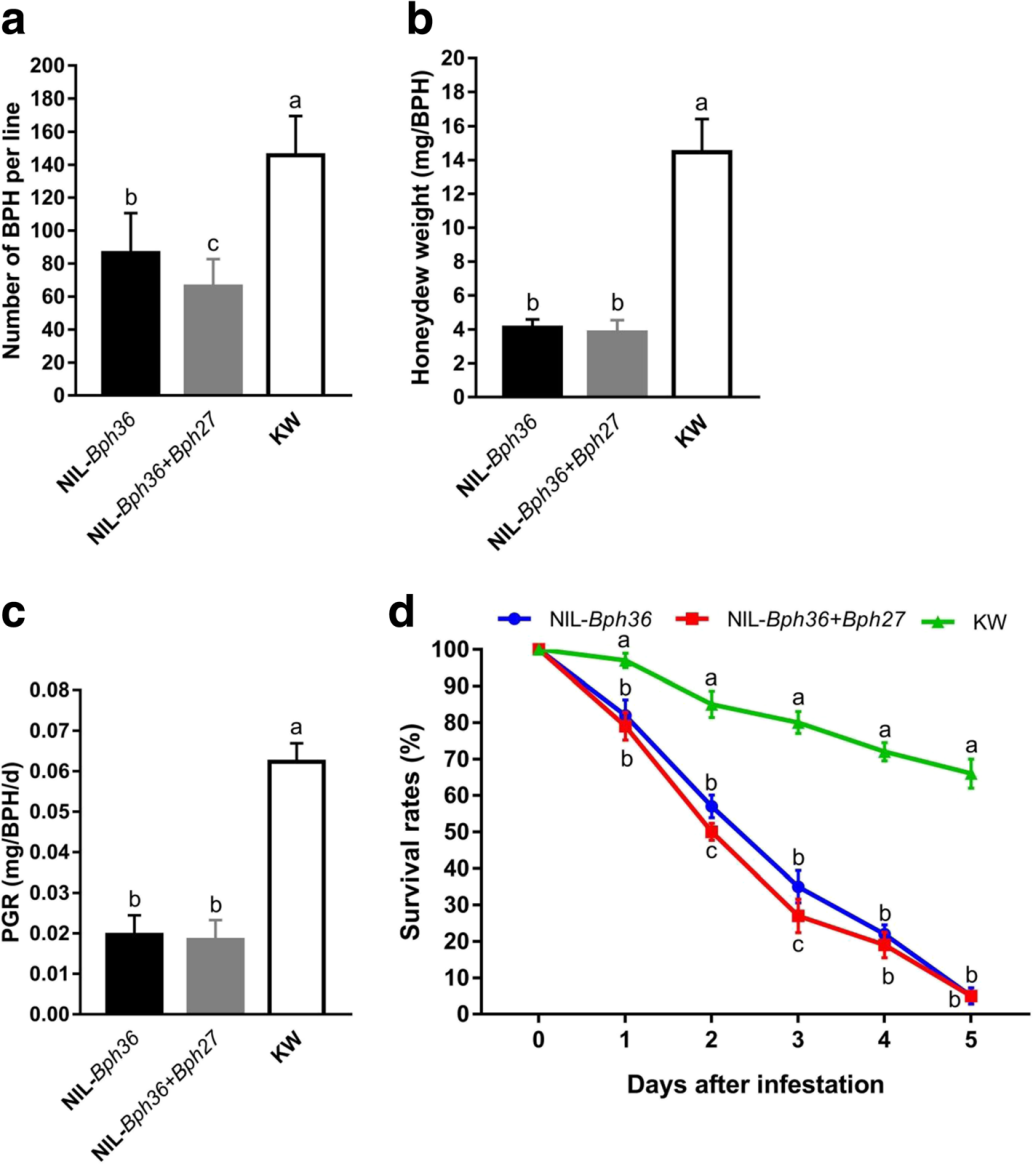
Characterization of the BPH resistance of NILs. **a** BPH host choice among NIL-*Bph36*, NIL-*Bph36* + *Bph27*, and KW. **b** Weight of honeydew secreted by BPH on NIL-*Bph36*, NIL-*Bph36* + *Bph27*, and KW. **c** Effect of NIL-*Bph36*, NIL-*Bph36* + *Bph27*, and KW on the BPH PGR (mg/BPH/d). **d** Survival rates of BPH on NIL-*Bph36*, NIL-*Bph36* + *Bph27*, and KW. Error bars represent SEs. Means labeled with the same letter are not significantly different at the level of *P* = 0.05 for *A. means* labeled with asterisks are significantly different (*P* < 0.05) for b and c. Statistical analysis was conducted with one-way ANOVA with LSD test

We characterized the BPH resistance of NIL-*Bph36* and NIL-*Bph36* + *Bph27*. In the BPH host choice test, the average number of BPHs landing on KW was measured as 145.7, while BPHs landing on NIL-*Bph36* + *Bph27* and NIL-*Bph36* were measured as 66.0 and 86.3, respectively. We observed a significant reduction in the number of settled BPHs between the resistant and susceptible lines (*P* < 0.001 for NIL-*Bph36* and KW or NIL-*Bph36* + *Bph27* and KW). Moreover, the number of BPHs settled on NIL-*Bph36* + *Bph27* was found to be significantly lower than that of NIL-*Bph36* (*P* < 0.05) (Fig. 4a). These results indicate that the resistance lines exhibited considerable antixienosis to BPH, and that the lines with two resistance genes exhibited stronger resistance to BPH.

According to our BPH honeydew excretion measurements for the different genotypic plants, we observed 14.46, 4.10, and 3.82 mg/BPH in KW, NIL-*Bph36*, and NIL-*Bph36* + *Bph27*, respectively (Fig. 4b). To measure resistance effects on the insects, the PGR of BPH was calculated from changes in weight observed before and after feeding the resistant and susceptible plants. Consequently, the PGRs of resistant plants NIL-*Bph36* and NIL-*Bph36* + *Bph27* and of susceptible plants KW were measured as 0.0196, 0.0184, and 0.0623 mg/BPH/d, respectively (Fig. 4c). Significantly less honeydew excretion levels and PGRs were detected from the resistant plants than from the susceptible plants (*P* < 0.001 for NIL-*Bph36* and KW or NIL-*Bph36* + *Bph27* and KW).

We surveyed the survival number of BPHs left 1 d, 2 d, 3 d, 4 d, and 5 d after treatment. BPH survival rates declined quickly for resistant plants NIL-*Bph36* and NIL-*Bph36* + *Bph27* compared to those of the susceptible plants. After 2 d of infestation, BPH survival rates for the resistant plants were less than 60%while remaining at above 80% for the susceptible plants (*P* < 0.05 for NIL-*Bph36* and KW or NIL-*Bph36* + *Bph27* and KW). After 5 d of infestation, BPH survival rates of the resistant plants were only measured at 5%, but a value of 62% was measured for the susceptible plants (*P* < 0.01 for NIL-*Bph36* and KW or NIL-*Bph36* + *Bph27* and KW, Fig. 4d). We must also note that survival rates of NIL-*Bph36* + *Bph27* were still lower than those of NIL-*Bph36* during the observation period, and a statistical difference was detected 2 d and 3 d after infestation (*P* < 0.05). Taken together the resistant plants had a strong antibiotic effect on the insects and the pyramided line had a stronger effect than the single gene line.

### BPH resistance evaluation for PYLs

To select PYLs for advanced breeding application, the developed lines were primarily evaluated through a BPH resistance test and from agronomic trait observations. Highly resistant PYLs (BC_3_F_3_) derived from RBPH16/RBPH54 × RBPH16/PTB33 with normal seed settings and heading dates were chosen for BPH resistance gene transformation. A total of seven PYLs were selected and applied to screen with the linked markers and to evaluate BPH resistance levels. Of these, lines VP1720, VP1728, VP1731, VP1732, and VP1733 were found to carry four resistant genes. Specifically, VP1720 and VP1728 were homozygous for the four resistance genes with average resistance scores of 1.76 and 1.59, respectively. VP1732 and VP1733 scored 2.73 and 2.18, respectively, and were found to be homozygous for three resistance genes and heterozygous for one gene. VP1731 was homozygous for *Bph36* and *Bph27* and heterozygous for the other two genes with an average BPH resistance score of 2.53. VP1722 and VP1734 were homozygous for three resistance genes and also exhibited considerable resistance to the BPH insects (Table 3). Therefore, all of the selected PYLs can be used for BPH resistance transfers for breeding application, especially for lines VP1720 and VP1728.

**Table 3:** Selected PYLs carring multiple BPH resistance genes

ID	X17^a^	RM16846	BYL18	RH007	Resistant score
VP1728	A^b^	A	A	A	1.59 ± 0.41
VP1720	A	A	A	A	1.76 ± 0.32
VP1733	A	A	H	A	2.18 ± 0.26
VP1722	A	A	A	B	2.53 ± 0.68
VP1731	A	A	H	H	2.53 ± 0.81
VP1732	A	A	H	A	2.73 ± 0.22
VP1734	A	A	B	A	2.95 ± 0.18

^a^X17, RM16846, BYL18, and RH007 were the linked molecular markers of *Bph36*, *Bph27*, *Bph29*, and *Bph3*, respectively. ^b^ A, B, and H denoted that the lines were homozygous for resistance and susceptible parents or heterozygous at the resistance gene region, respectively

### Breeding applications of resistance genes

**Fig. 5:**
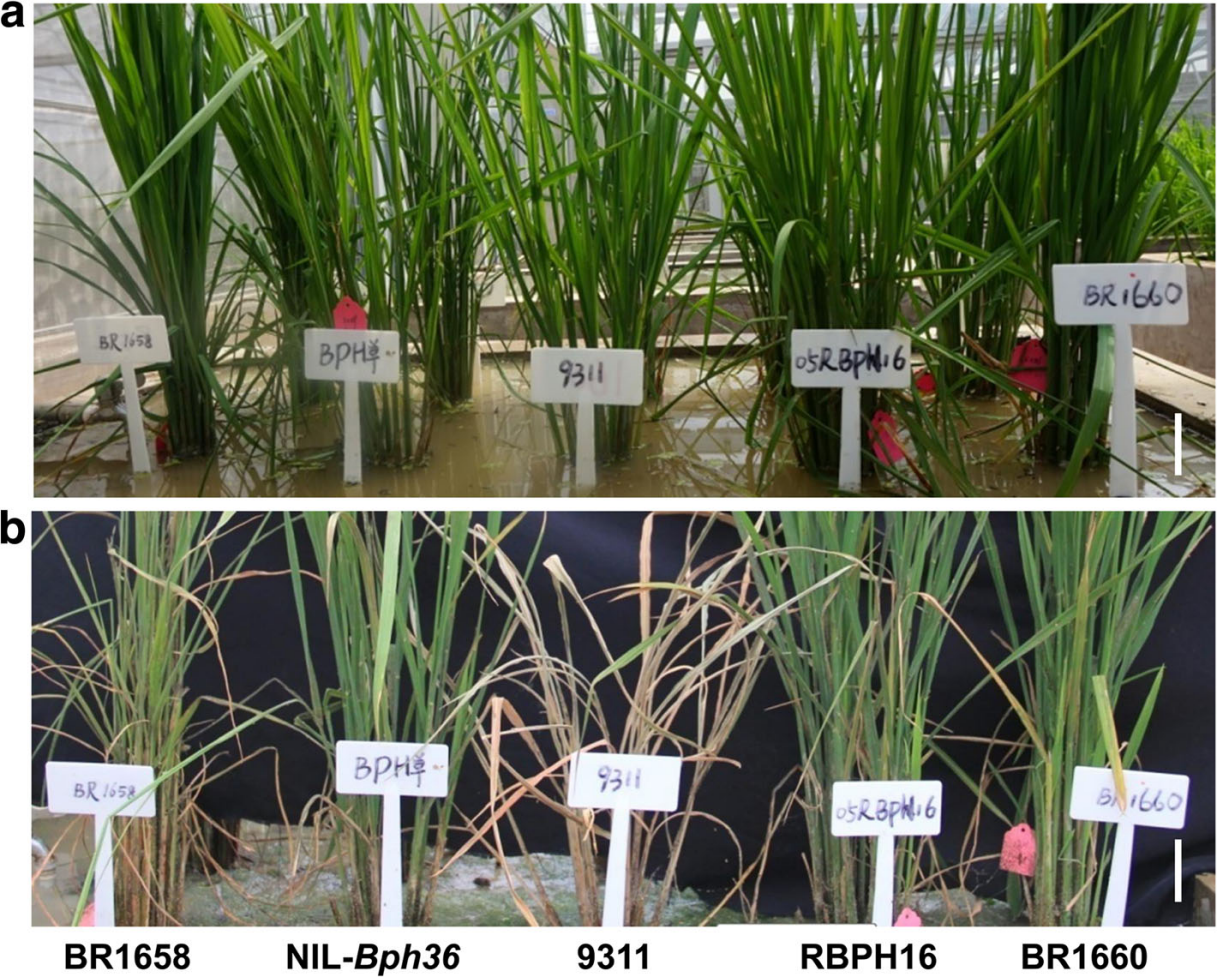
BPH resistance test at tillering stage. **a** Before infestation with BPH, **b** After infestation with BPH. RBPH16 and 9311 were the resistance and susceptible controls, respectively. BR1658 and BR1660 had *Bph36*, *Bph27*, *Bph29*, *Bph3*, and *Xa23* with MH511 genetic background and were indicated to be highly resist to BPH. NIL-*Bph36* carried single gene *Bph36* with KW genetic background. Scale bars, 15 cm

**Fig. 6:**
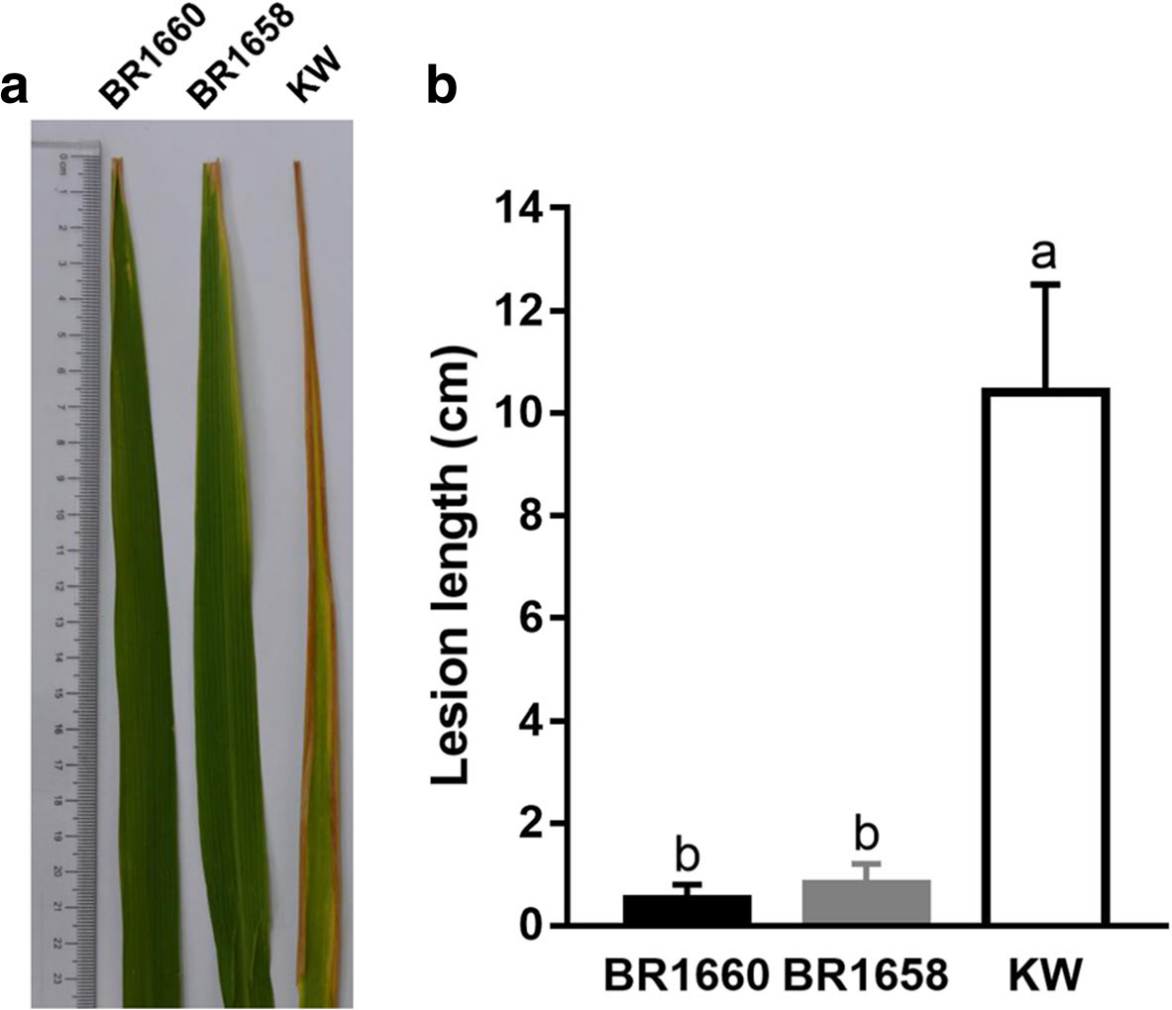
Test for bacterial blight resistance of PYLs at tillering stage. **a** Bacterial blight resistance of BR1658, BR1660 and KW to GX1070 using the leaf-clipping method. Leaf lesions 2 weeks post inoculation are indicated by representative leaves. **b** Lesion length measurement. Error bars represent SEs. Means labeled with the same letter are not significantly different at the level of *P* = 0.05. Statistical analysis was conducted with one-way ANOVA with LSD test

To develop breeding lines with strong BPH resistance and merit agronomic traits, the selected PYL of VP1728 was continuously backcrossed with 9311 and MH511. As a result, rice lines BR1658 and BR1660 carrying *Bph36*, *Bph27*, *Bph29*, *Bph3*, and *Xa23* with an MH511 genetic background were found to be highly resistant to BPH with average resistance scores of 2.93 and 2.12 at the seedling stage, respectively. They also exhibited strong resistance to BPH insects in the tillering stage (Fig. 5). Both lines were then also found to be highly resistant to bacterial blight in the tillering stage (Fig. 6). Average disease infection lengths were measured as 0.6, 0.5, and 10.2 cm for BR1658, BR1660, and KW, respectively (*P* < 0.001 for BR1658 and BR1660 relative to KW). Rice line BR1693 carrying *Bph36*, *Bph27*, *Bph3* and *Bph29* with the 9311 genetic background also exhibited strong resistance to BPH insects with an average resistance score of 2.14 for the seedling stage.

To evaluate the developed breeding lines, we surveyed agronomic traits of BR1658, BR1660, and BR1693 and of recurrent parents MH511 and 9311. The plant height (106.9 cm), number of effective panicles (8.5), and setting rate (84.0) of BR1693 were found to be less than those of 9311, and the number of effective panicles (176.8) was found to be greater than that of 9311. Howerer, we found that no statistical difference between BR1693 and 9311 (*P* > 0.05). Interestingly, the average 1000-grain weight of BR1693 was measured as 25.4 g, which is lower than that found for 9311 (27.8 g) (*F* = 16.4, *P* = 0.042, Table 4). As for rice lines BR1658 and BR1660, all the detected traits were identical to those of MH511 (*P* > 0.05). Although the setting rates of BR1658 (85.1%) and BR1660 (84.8%) decreased relative to those of MH511 (86.4%) (*F* = 5.6, *P* = 0.06 for BR1658 and MH511; *F* = 4.8 *P* = 0.046 for BR1660 and MH511), no statistical difference was observed between them (Table 4). These results indicate that the developed breeding lines acquired high BPH and disease resistance values and exhibited the same agronomic traits as their recurrent parents.

**Table 4:** Agronomic traits of recurrent parents and breeding lines

Lines^a^	Plant height (cm)	Number of effective panicle per plant	Number of grains per panicle	Setting rate (%)	1000-grain weight (g)
9311	108.6 ± 0.70	8.7 ± 0.37	168.9 ± 5.06	84.7 ± 1.53	27.8 ± 0.35
BR1693	106.9 ± 0.92	8.5 ± 0.44	176.8 ± 4.15	84.0 ± 1.28	25.4* ± 0.16
MH511	96.4 ± 2.21	6.9 ± 0.57	141.6 ± 5.06	86.4 ± 2.15	21.3 ± 0.23
BR1658	95.4 ± 1.15	7.0 ± 0.42	139.7 ± 7.46	85.1 ± 1.56	21.6 ± 0.37
BR1660	94.9 ± 1.76	6.8 ± 0.52	141.8 ± 5.24	84.8 ± 1.72	20.9 ± 0.68

^a^9311 and MH511 were the susceptible parents; BR1693 was the highly BPH resistance line with 9311 genetic background; BR1658 and BR1660 were the highly BPH and bacterial blight resistance lines with MH511 genetic background. *P < 0.05 derived from one-way ANOVA with LSD test

## Discussion

BPHexerts chronic biotic stress and severely constrains rice production in Asia and in other parts of the world. Accordingly, BPH resistance gene identification, mapping and characterization are valuable in ensuring long-term resistance against diverse predominant biotypes for sustainable rice outputs. Previous reports demonstrate the highly resistant wild rice accession GX2183 contains more than one major gene for BPH resistance. In this study, RBPH16 and RBPH17 originating from GX2183 were employed as donor parents and were respectively crossed to KW and HHZ to develop F_2:3_ mapping populations. Subsequently, with the exception of the previously detected gene *Bph27* (Huang et al. 2013), novel resistance gene *Bph36* was identified via high-resolution mapping to accupy a 38-kb region harbored by markers S13 and X48. Rice chromosomes 4 and 12 have been considered as the mainframe of BPH resistance genes (Jena and Kim 2010; Fujita et al. 2013; Hu et al., 2016a, b). For instance, thirteen genes are found in two distinct clusters of chromosome 4. Six of them (*Bph6*, *bph12*, *Bph27, bph18*(*t*)*, Bph27*(*t*)*,* and *Bph34*) are clustered on the long arm of chromosome 4 (Qiu et al. 2010, 2012; Huang et al. 2013; Fujita et al. 2013; Kumar et al. 2018); and seven genes (*Bph3, Bph12, Bph15, Bph17, Bph20*(*t*), *Bph30*, and *Bph33*) are found on the short arm of chromosome 4 (Liu et al., 2015; Yang et al. 2002; Sun et al. 2005; Yang et al. 2004; Rahman et al. 2009; Wang et al. 2018; Hu et al. 2018). According to the present study *Bph36* also occupies this cluster, and via high-resolution mapping, it was successfully identified on the short arm of chromosome 4 roughly 2 Mb downstream from the *Bph12, Bph17*, *Bph15*, and *Bph20* regions (Yang et al. 2002; Sun et al. 2005; Yang et al. 2004; Rahman et al. 2009) and 0.6 Mb upstream from *Bph3* in the Nipponbare genome (Liu et al., 2015). Gene *Bph36* presented a large LOD score and accounts for the considerable variance in BPH resistance observed as shown in Table 1. This was also proved through the characterization of NILs and PYLs as shown in Figs. 4 and Fig. 5.

Plants generally apply antixenosis, antibiosis, and tolerance against insect attacks. Previous studies indicate that BPH resistance genes such as *Bph6*, *Bph14*, and *Bph27* have strong antixenotic and antibiotic effects on the insects (Qiu et al. 2010; Du et al. 2009; Huang et al. 2013), while the resistance gene *Bph7* mainly confers tolerance to BPH (Qiu et al. 2014). Moreover, plants pyramiding with two or more genes significantly increase resistance to BPH insects (Qiu et al. 2012; Hu et al. 2012). According to tests on BPH host choices, honeydew excretion, survival rates, and PGR, lines of NIL-*Bph36* and NIL-*Bph36* + *Bph27* show significant antixenotic and antibiotic effects on insects relative to those of susceptible plants, and it must be noted that lines of NIL-*Bph36* + *Bph27* have significantly stronger resistance effects than NIL-*Bph36* as shown in host choice and BPH survival rates (Fig. 4).

Wild rice species offer natural and unique pools of resistance genes with strong resistance to all BPH biotypes (Heinrichs et al. 1982; Hu et al. 2015). At the same time, modern technologies offer means of using molecular markers in molecular breeding approaches to accelerate the identification and application of resistance loci (Ashkani et al. 2015). Recently, Jiang et al. (2018) introduced six dominant BPH-resistance genes into Jin23B using MAS and in turn improved BPH-resistance breeding lines. Moreover, Hu et al. (2016a, b) pyramided *Bph3*, *Bph14*, and *Bph15* into elite *indica* cultivar Heimeizhan and obtained high BPH resistance breeding lines. Via high-resolution mapping, *Bph36* has been found to include tightly linked markers that are effective and efficient for MAS performance (Additional file 2: Figure S2). We also pyramided *Bph36* with *Bph27*, *Bph29*, and *Bph3* by continuous backcrossing and MAS, and obtained several highly resistant PYLs. Of these, VP1720 and VP1728 were found to be homozygous across all four BPH-resistance genes and exhibited strong resistance to insects (Table 3). Then, VP1728 was backcrossed with MH511 and 9311 to develop approximate resistant breeding lines with diverse backgrounds. Consequently, an agronomic trait survey suggests that the developed breeding lines are almost the same as the recurrent parents (Table 4). Therefore, the developed PYLs may be applied for insect resistance rice breeding.

Regarding agricultural traits of the developed PYLs, setting rates of BR1658 (85.1%) and BR1660 (84.8%) declined relative to those of MH511 (86.4%) (*F* = 5.6, *P* = 0.06 for BR1658 and MH511; *F* = 4.8 *P* = 0.046 for BR1660 and MH511). The 1000-grain weight of BR1693 (25.4 g) was also lower than that of 9311 (27.8 g) (*F* = 16.4, *P* = 0.042, Table 4). This suggests that linkage drag remains a serious problem when pyramiding different genes via MAS. Many previous studies have revealed the same problem. For instance, a previous study shows that *Bph3* from Rathu Heenati is positioned very close to the *Wx*^*a*^ allele (roughly 380 kb), which confers amylose content in rice (Jairin et al. 2007, 2009). However, the authors acquired breeding lines with *Bph3* and without *Wx*^*a*^ alleles by screening large backcrossed individuals (Jairin et al. 2009). However, Hu et al. (2016a, b) failed to break up linkages of *Wx*^*a*^ while pyramiding *Bph3* with *Bph14* and *Bph15* in Hemeizhan, as the numbers of screened backcrossed individuals was still too low. Therefore, more backcrosses and MAS tests are needed to break up unexpected linkage genes for breeding purposes.

Our results validate the usefulness of the *Bph36* gene for the control of BPH and show that applying it to susceptible rice varieties produces resistance effect by suppressing the feeding behavior, growth and longevity of BPH insects. This can in turn facilitate the development of BPH-resistant rice varieties in the future and help limit herbicide use. Tightly linked markers will facilitate the marker-assisted breeding and high-resolution mapping of *Bph36*, laying the foundation for insect resistance mechanism research, map-based cloning and functional analysis and providing a theoretical basis and intermediate materials to facilitate resistance to BPH.

## Additional files

Additional file 1: **Figure S1.** The pedigree of pyramiding lines (PYLs) containing resistance genes *Bph36*, *Bph27*, *Bph29*, *Bph3*, and *Xa23* developed using marker-assisted selection (MAS). (TIF 359 kb)

Additional file 2: **Figure S2** Amplified bands of BC_1_F_2_ individuals derived from KW/RBPH16 with InDel marker X17 and detected by 7% PAGE. M: Marker DL2000, RBPH16: resistance parent, KW: susceptible parent, Lanes 1–22: individuals of BC_1_F_2_. (TIF 638 kb)

Additional file 3: **Table S1** Markers for marker-assisted selection (MAS) of BPH and bacterial blight resistance genes. (DOCX 16 kb)
