# Supplementary figures and images for: High-resolution mapping and breeding application of a novel brown planthopper resistance gene derived from wild rice (Oryza. rufipogon Griff)
Source: Rice (N Y). 2019 Jun 4;12:41. doi: 10.1186/s12284-019-0289-7 (PMC6548798; doi:10.1186/s12284-019-0289-7)

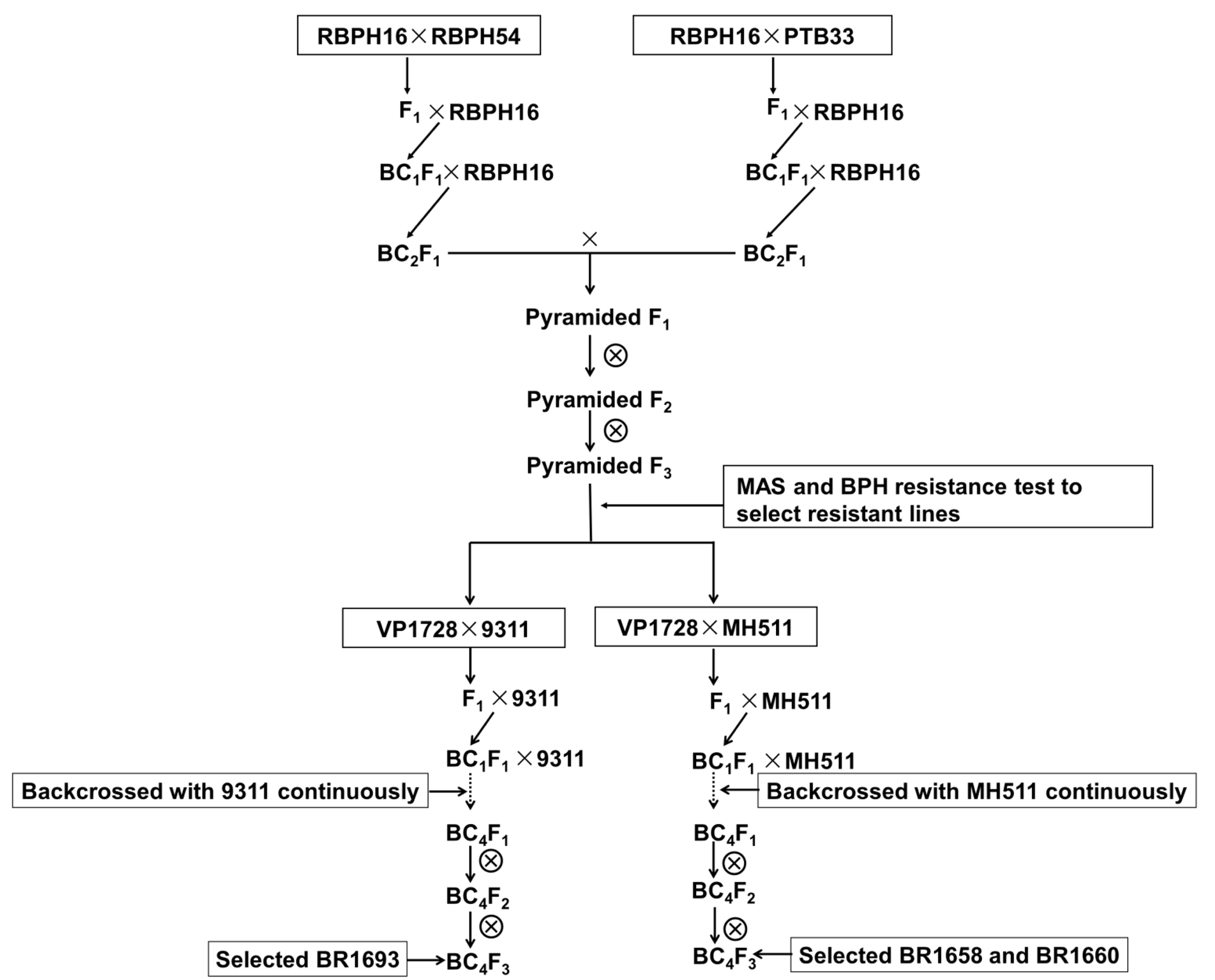

Supplement: Supplementary file 1 — Figure S1. The pedigree of pyramiding lines (PYLs) containing resistance genes Bph36, Bph27, Bph29, Bph3, and Xa23 developed using marker-assisted selection (MAS). (TIF 359 kb) [file 12284_2019_289_MOESM1_ESM.tif]

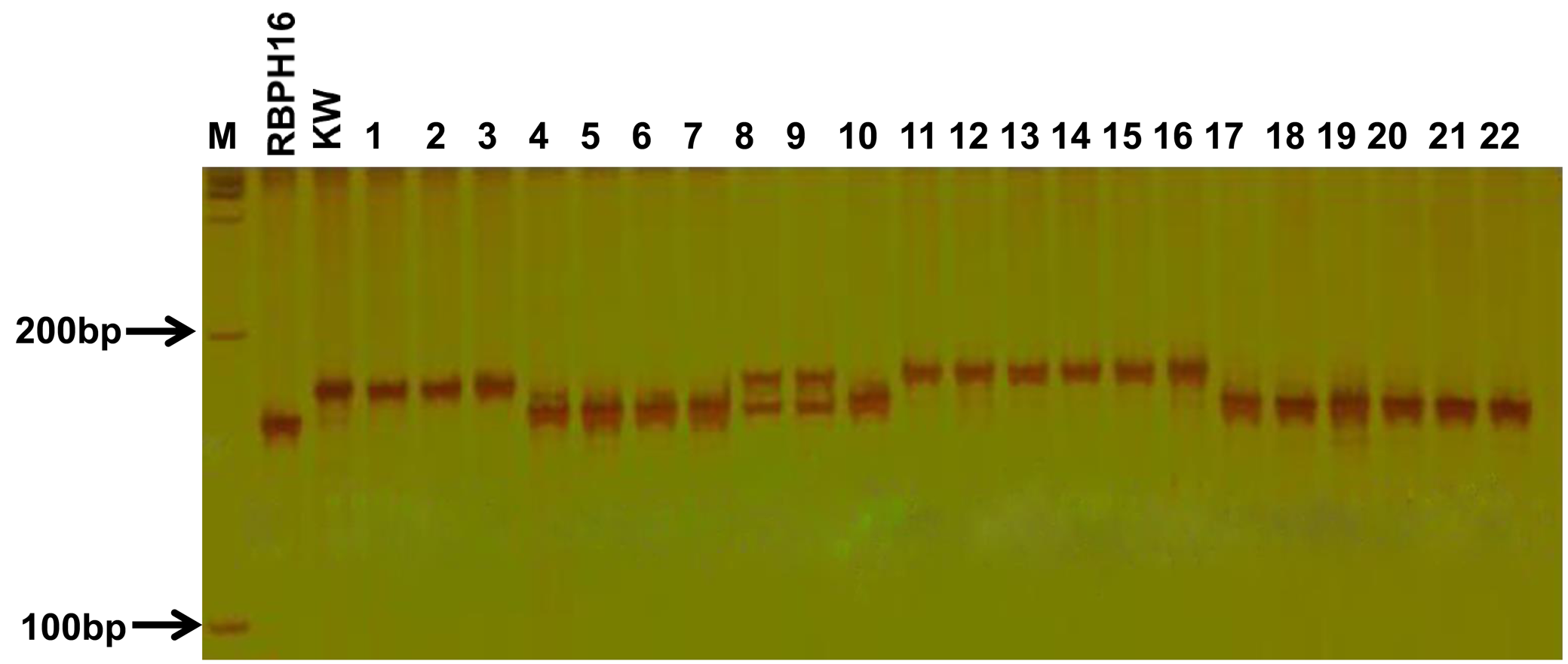

Supplement: Supplementary file 2 — Figure S2 Amplified bands of BC1F2 individuals derived from KW/RBPH16 with InDel marker X17 and detected by 7% PAGE. M: Marker DL2000, RBPH16: resistance parent, KW: susceptible parent, Lanes 1–22: individuals of BC1F2. (TIF 638 kb) [file 12284_2019_289_MOESM2_ESM.tif]
