# Supplementary material for: High-resolution mapping and breeding application of a novel brown planthopper resistance gene derived from wild rice (Oryza. rufipogon Griff)
Source: Rice (N Y). 2019 Jun 4;12:41. doi: 10.1186/s12284-019-0289-7 (PMC6548798; doi:10.1186/s12284-019-0289-7)
Supplement: Supplementary file 3 — Table S1 Markers for marker-assisted selection (MAS) of BPH and bacterial blight resistance genes. (DOCX 16 kb) [file 12284_2019_289_MOESM3_ESM.docx]

**Table S1** Markers for marker-assisted selection (MAS) of BPH and bacterial blight resistance genes

| Gene | Marker^1^ | Forward primer (5'-3') | Reverse primer (5'-3') |
| --- | --- | --- | --- |
| *Bph3* | RH007 | CTTGCGTTCCGTAGGAGAAG | TGAGTGTAACCCGAAGTGGC |
| *Bph27* | RM16846 | CTACAAGCAACACAGTATCACAGC | GGTAACTGGTGCTTATTTAGCC |
| *Bph29* | BYL18 | CCCACTTCCACAACCACA | ATGCTCCTAGCTTCCTATTCC |
| *Xa23* | Lj36 | GCAATGGCTAGTAGGAACGA | ATCCGCACAAGAACAGTAGC |

^1^The linked markers RH007, RM16846, BYL18, and Lj36 were referred Liu et al. (2014), Huang et al. (2013), Wang et al. (2015), and Wang et al. (2014), respectively.
